# Supplementary material for: Lactone Stabilized by Crosslinked Cyclodextrin Metal-Organic Frameworks to Improve Local Bioavailability of Topotecan in Lung Cancer
Source: Pharmaceutics. 2022 Dec 31;15(1):142. doi: 10.3390/pharmaceutics15010142 (PMC9865350; doi:10.3390/pharmaceutics15010142)
Supplement: Supplementary file 1 [file pharmaceutics-15-00142-s001.zip › pharmaceutics-2035254-supplementary.pdf]

Supporting Information

Lactone stabilized by crosslinked cyclodextrin metal-organic frameworks to improve local bioavailability of topotecan in lung cancer

Ting Xiong [1,2,5], Tao Guo [2], Yaping He [3], Zeying Cao [2,4], Huipeng Xu [2,4], Wenting Wu[1], Li Wu [2], Weifeng Zhu [1*] and Jiwen Zhang [1,2,5*]

Author affiliations:

[1]Key Laboratory of Modern Preparation of TCM, Ministry of Education, Jiangxi University of Traditional Chinese Medicine, Nanchang, 330004, China

[2]Center for Drug Delivery Systems, Shanghai Institute of Materia Medica, Chinese Academy of Sciences, Shanghai, 201203, China

[3]Department of Pharmacy, The First Affiliated Hospital of Zhengzhou University, Zhengzhou, 450052, China

[4]University of the Chinese Academy of Sciences, Beijing, 100049, China

[5]College of Pharmacy, Shenyang Pharmaceutical University, Shenyang, Liaoning 110016, China

Corresponding Author:

Center for Drug Delivery Systems, Shanghai Institute of Materia Medica, Chinese Academy of Sciences, Shanghai, 201203, China; Tel/Fax: +86-21-20231980; E-mail: jwzhang@simm.ac.cn (Prof. Jiwen Zhang)

Key Laboratory of Modern Preparation of TCM, Ministry of Education, Jiangxi University of Traditional Chinese Medicine, Nanchang, 330004, China; Tel/Fax: +86-791-87118614; E-mail: zwf0322@126.com (Prof. Weifeng Zhu)

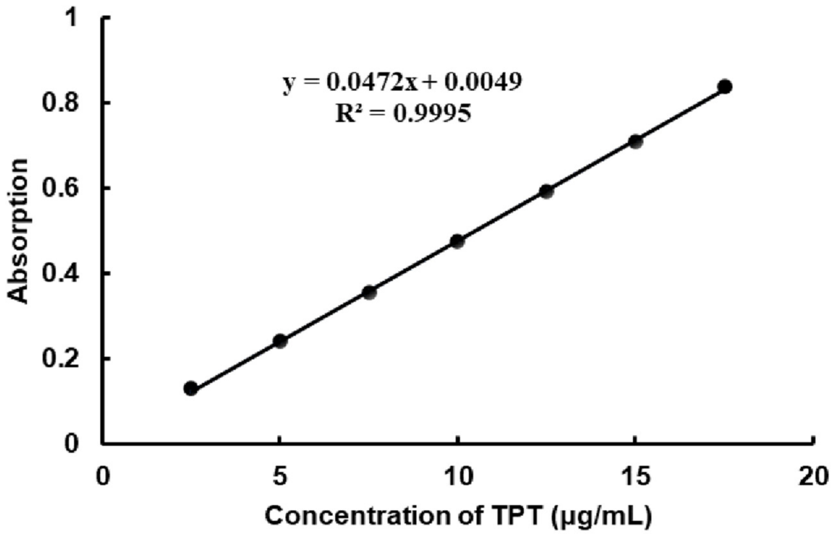

Figure S1. Linear calibration curve of the standard TPT solution.

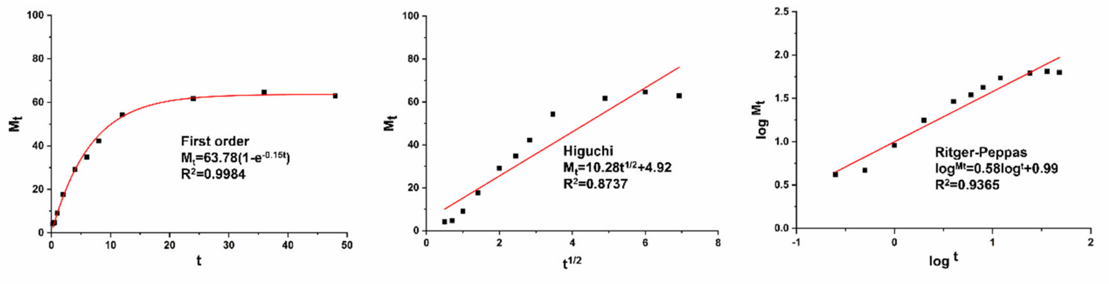

Figure S2. The *in vitro* release profile of TPT@CL-MOF was fitted by first-order kinetics, Higuchi models and Ritger-Peppas models. M_t is the amount of drug released at time t.

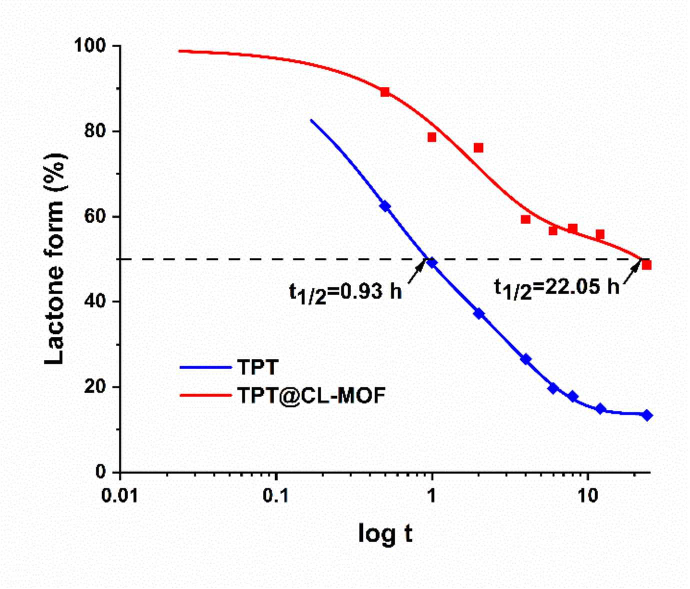

Figure S3. Degradation data of TPT and TPT@CL-MOF were fitted on biexponential decay equation in PBS (pH 7.4). $t_{1/2}$ is the half-life of TPT against hydrolysis.

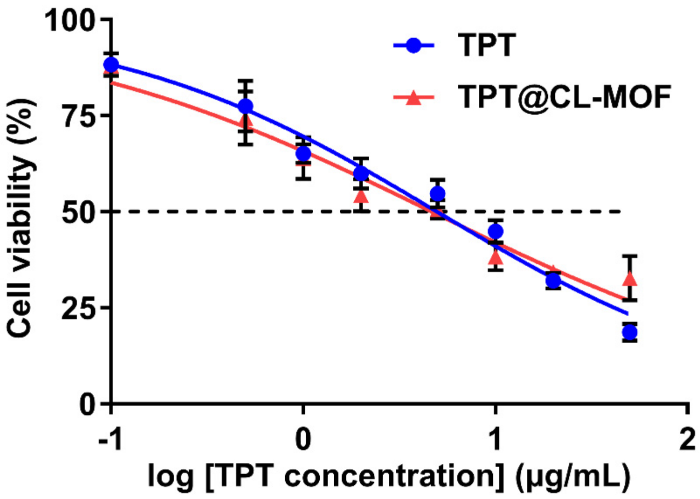

Figure S4. The IC_{50} of TPT and TPT@CL-MOF against B16F10 cells.

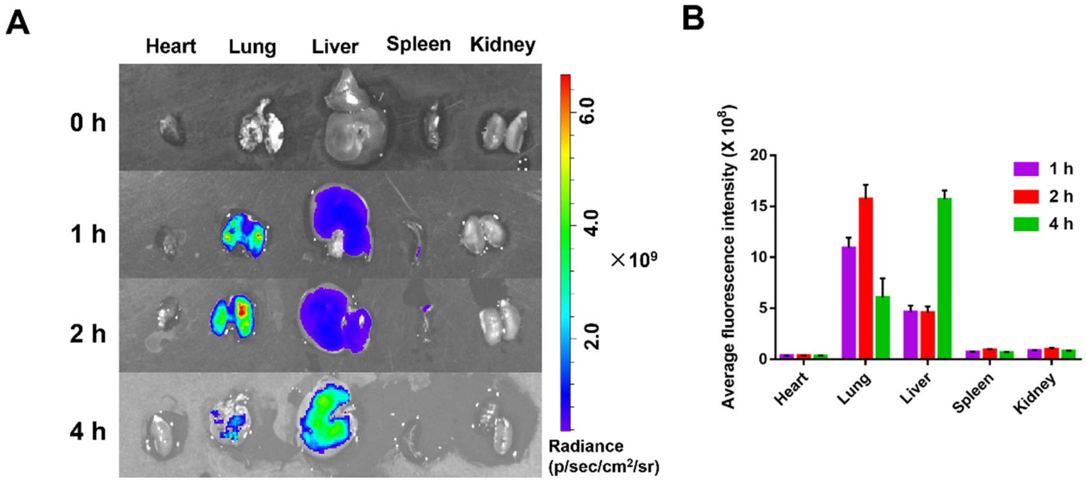

Figure S5. Tissue distributions of TPT@CL-MOF in C57BL/6 mice (A) and semi-quantitative analysis (B) (n = 3).

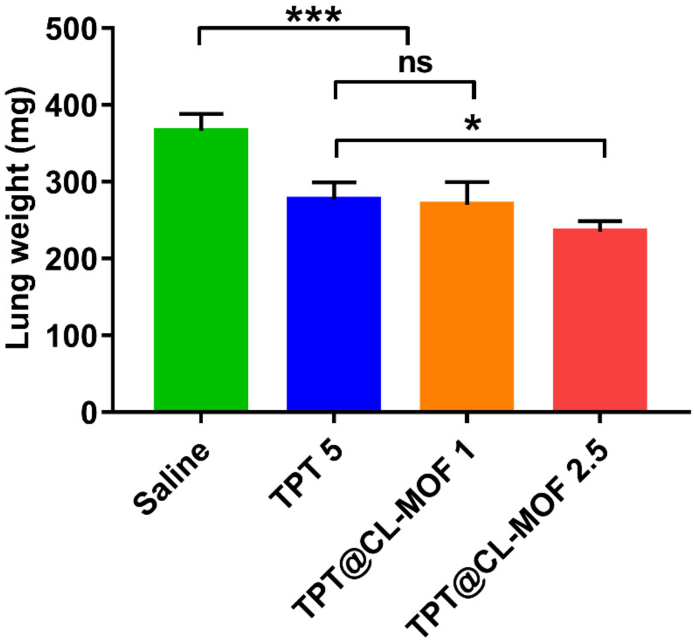

Figure S6. The weight of lung tissues isolated from the C57BL/6 mice bearing B16F10 cells (n = 5). TPT@CL-MOF 1 and TPT@CL-MOF 2.5 denotes TPT at 1 mg/kg and 2.5 mg/kg, respectively. *$p < 0.05$, ***$p < 0.001$. The abbreviation "ns" denotes no significant difference between relevant treatment groups.
